# Host reproductive cycle influences the pouch microbiota of wild southern hairy-nosed wombats (*Lasiorhinus latifrons*)

**DOI:** 10.1186/s42523-021-00074-8

**Published:** 2021-01-25

**Authors:** Sesilje Weiss, David Taggart, Ian Smith, Kristofer M. Helgen, Raphael Eisenhofer

**Affiliations:** 1 Department of Veterinary and Animal Sciences, University of Copenhagen, Frederiksberg C, Denmark; 2 School of Animal and Veterinary Sciences (Waite), University of Adelaide, Adelaide, South Australia Australia; 3 FAUNA Research Alliance, Ltd, PO Box 98, Callaghan, NSW 2308 Australia; 4 Zoos South Australia, Frome Rd, Adelaide, South Australia Australia; 5 Australian Museum Research Institute, 1 William St, Sydney, New South Wales Australia; 6 Australian Research Council Centre for Australian Biodiversity and Heritage, University of New South Wales, Sydney, New South Wales Australia; 7 School of Biological Sciences, University of Adelaide, Adelaide, South Australia Australia; 8 Australian Research Council Centre for Australian Biodiversity and Heritage, University of Adelaide, Adelaide, South Australia Australia

## Abstract

**Background:**

Marsupials are born much earlier than placental mammals, with most crawling from the birth canal to the protective marsupium (pouch) to further their development. However, little is known about the microbiology of the pouch and how it changes throughout a marsupial’s reproductive cycle. Here, using stringent controls, we characterized the microbial composition of multiple body sites from 26 wild Southern Hairy-nosed Wombats (SHNWs), including pouch samples from animals at different reproductive stages.

**Results:**

Using qPCR of the 16S rRNA gene we detected a microbial community in the SHNW pouch. We observed significant differences in microbial composition and diversity between the body sites tested, as well as between pouch samples from different reproductive stages. The pouches of reproductively active females had drastically lower microbial diversity (mean ASV richness 19 ± 8) compared to reproductively inactive females (mean ASV richness 941 ± 393) and were dominated by gram positive bacteria from the Actinobacteriota phylum (81.7–90.6%), with the dominant families classified as Brevibacteriaceae, Corynebacteriaceae, Microbacteriaceae, and Dietziaceae. Three of the five most abundant sequences identified in reproductively active pouches had closest matches to microbes previously isolated from tammar wallaby pouches.

**Conclusions:**

This study represents the first contamination-controlled investigation into the marsupial pouch microbiota, and sets a rigorous framework for future pouch microbiota studies. Our results indicate that SHNW pouches contain communities of microorganisms that are substantially altered by the host reproductive cycle. We recommend further investigation into the roles that pouch microorganisms may play in marsupial reproductive health and joey survival.

**Supplementary Information:**

The online version contains supplementary material available at 10.1186/s42523-021-00074-8.

## Background

Marsupials and eutherian (placental) mammals diverged evolutionarily approximately 160 million years ago [1], and although they are both mammals belonging to the same subclass, Theria, they have major distinguishing features, especially with regard to their reproductive biology. The most salient difference is the birth of the marsupial neonate (joey) at a much earlier stage of development compared to placental mammals. Birth of the joey generally occurs after an extremely short gestation period of 12–38 days [2], after which the joey crawls toward and attaches to a teat for further nourishment and development, usually in the safety of an external pouch (marsupium). This early exposure of the joey—with its underdeveloped immune system—to the environment poses a challenge as microbial infections could lead to joey mortality. The marsupial pouch provides an enclosed physical space separate to the environment, and facilitates the passive transfer of antimicrobial peptides and immunoglobulins to the joey through milk which are believed to play a significant role in the immune protection during their pouch life [3, 4]. In addition, it is thought that changes to the pouch environment itself occur through pouch secretions, which could in turn influence pouch microbial community composition [4–6], perhaps to a protective state.

In general, the microbiome of the female reproductive system remains less well characterized than the microbial complement of the gut, and this is especially so for marsupials. Earlier, culture-based studies on koala (*Phascolarctos cinereus*), quokka (*Setonix brachyurus*), and Tammar wallaby (*Notamacropus eugenii*) pouches found substantial reductions or the absence of viable bacteria isolated from the pouches of mothers leading up to birth [7–10]. Studies using molecular techniques on Tammar wallaby and brushtail possum (*Trichosurus vulpecula*) pouches found changes in microbial composition associated with reproductive stage [11, 12]. To date, there have only been two NGS (next-generation sequencing) studies on marsupial pouches, both on the Tasmanian devil, *Sarcophilus harrisii* [13, 14]. Cheng et al. reported that pouch microbial composition from 18 non-lactating devils was similar to that of skin [13], and Peel et al. reported differences in microbial composition between the pouches of three lactating and three non-lactating devils [14]. If and how microbes in the marsupial pouch influence the reproductive success of the host remains to be determined, however, there is increasing awareness that DNA contamination can confound NGS studies that target sample types with low microbial biomass [15, 16]. Therefore, it is crucial to apply a robust authentication framework when exploring the microbiome of new sample types, or those that have been understudied such as the marsupial pouch [16].

To further investigate the microbial component of the pouch environment, and to create a robust framework for future pouch microbiome research, we studied the Southern Hairy-nosed Wombat (SHNW), *Lasiorhinus latifrons*. The SHNW (SI figure 1) is a large (~ 23–38 kg for adults) nocturnal, burrowing marsupial herbivore which occurs in the semi-arid rangelands of southern South Australia and south-eastern Western Australia [17]. This species is a grassland specialist, feeding on native perennial grasses, principally *Stipa nitida* [18]*.* The SHNW has a home range estimated at around 1–5 ha, which is small for a herbivorous animal of its size [19, 20]. SHNWs breed between mid-July and December depending upon rainfall and forage availability, with most new births occurring between August–October [20, 21]. After a short gestation period of ~ 22 days, a single joey is born and crawls into a ventral-facing pouch containing two teats [22]. Joeys remain in the pouch until the following May and are weaned at approximately 1 year of age [23].

In this study, we characterized the microbial composition of different body sites of 26 wild female SHNWs, including oral, skin, pouch, milk, cloacal, and gut (faecal), with a primary focus on investigating differences in pouch microbial composition in relation to seasonal/reproductive stage. We hypothesized that the microbial diversity of the pouch would decrease in reproductively active females due to maternal protective mechanisms. We also developed and applied a stringent workflow to control for DNA contamination in pouch microbiome studies, which can form a solid framework for future pouch NGS studies.

## Methods

All research involving animal capture, handling and sample collection was carried out under University of Adelaide Animal Ethics Permit #S-2018-112 and South Australian Department of Environment and Water Scientific Research Permit #A26820–2.

### Study site and sampling period

**Fig. 1:**
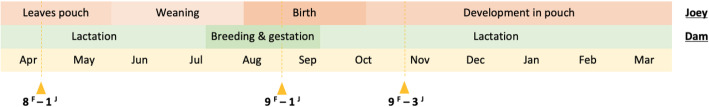
Timeline of the three field trips in relation to the typical reproductive cycle of female SHNWs and the developing young. Note that these reproductive timelines are averages, and that some variation can occur due to rainfall/food availability. Arrows indicate when the field trip took place, and the numbers indicate the number of female wombats (^F^) and joeys (^J^) caught and sampled

**Fig. 2:**
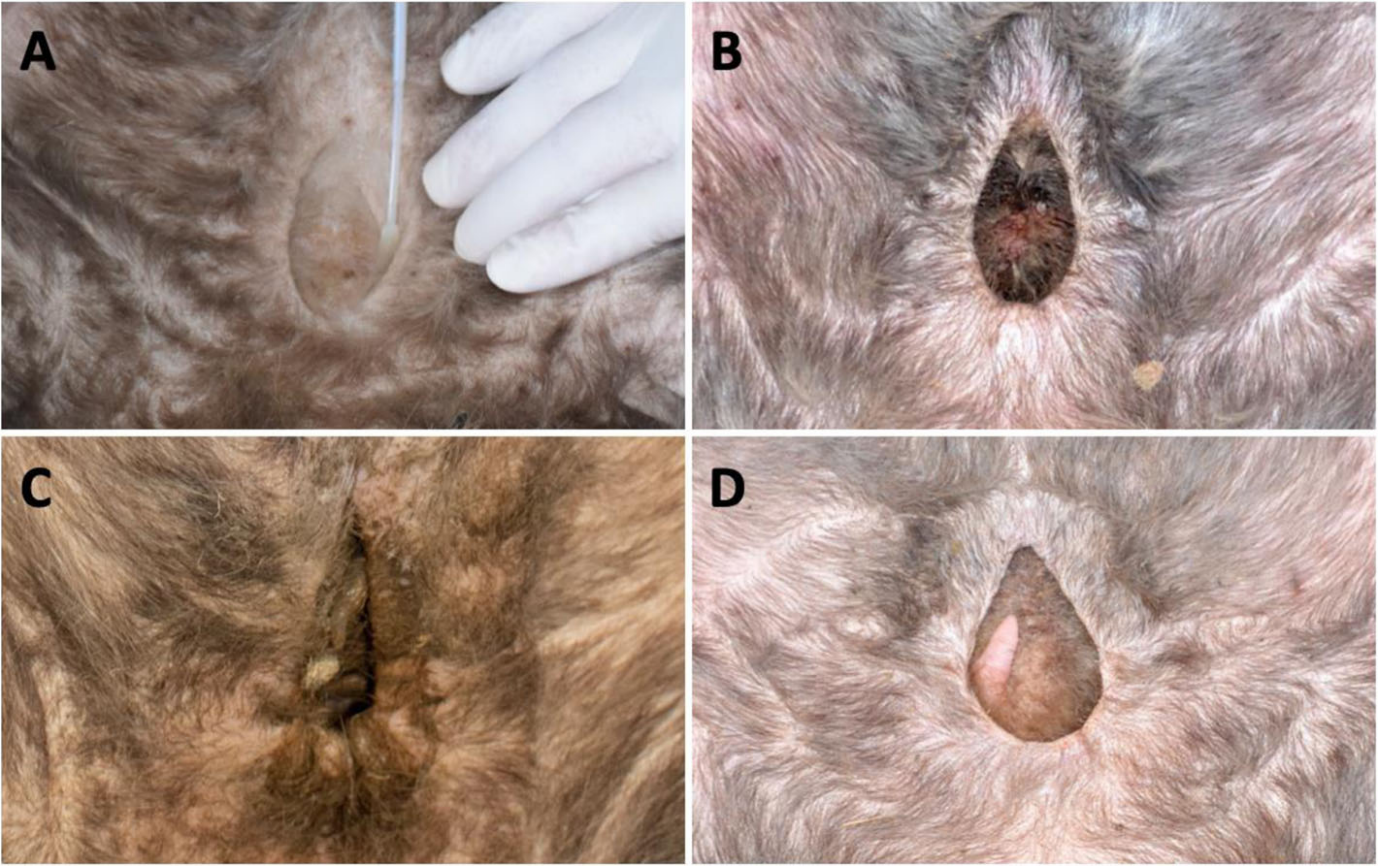
Southern Hairy-nosed Wombat pouches during different reproductive stages. A) Subadult pouch, characterised by the dry, clean and shallow appearance. B) Pouch of an adult female either in oestrus or during gestation, characterized by being moist with dark red coloured secretions, deep, and with a thick muscular pouch wall. C) Pouch of an adult female during lactation, with a 5.5 months old joey inside, characterized by being moist, dirty, deep and with a tight pouch entrance. D) Pouch of an adult female early post lactation / anoestrus, with a characteristic dry and deep appearance, with an enlarged, but regressing teat

The study was conducted at Kooloola Station near Swan Reach, 34°32′20.6″S 139°35′48.4″E, approximately 130 km northeast of Adelaide in South Australia’s Murraylands. All animals were captured within a 5 km radius of the station homestead. Field trips lasted 3–4 days and were conducted during 2019 (Fig. 1). Timing of the field trips was planned and conducted according to the SHNW breeding cycle to allow for sampling of females and pouch young throughout the different stages of pouch and pouch-young maturity (Fig. 2). Field trips were conducted in April, late August and October, which theoretically allowed for sampling of newborn joeys up to large mature pouch young, as well as newly weaned juveniles (Fig. 1).

### Sample collection

Wombats were captured at night in April, August and October 2019 using a spotlight and large custom made, hand-held wombat net [24]. Captured animals were placed in hessian sacks and returned to an old shearing shed for processing and microbial sample collection the following morning. All wombats were anaesthetized with an injection of zolazepam (50 mg/ml) + tiletamine (50 mg/ml), 3 mg total combined dose per kg delivered intramuscularly. (ZOLETIL® 100 VET; Virbac Animal Health, Milperra, New South Wales, Australia) prior to data and sample collection. Once anaesthetised all captured female wombats had microbial samples collected, and were then measured (weight, head length, width, pes length and body condition). It was only possible to classify gestating wombats and animals near oestrus to one collective group: cycling. This is due to lack of morphological differences between the two reproductive states [25], the lack of known pregnancy biomarkers and the infeasibility of repetitive blood sampling in the field. All microbial samples (except faecal) were collected using COPAN regular nylon FLOQSwabs (520CS01), which have been demonstrated to collect more microbial cells compared to cotton swabs [26]. To minimise and control for DNA contamination, the recommended guidelines proposed by Eisenhofer et al. [16] were followed.

To reduce the contamination of biological samples from the people undertaking sampling, all persons involved in sampling wore facemasks, gloves, and sleeves to cover arms. Samples were taken in the order of; mouth, skin, pouch, cloaca, and faecal, and gloves were changed between animals and in the event of becoming contaminated by the previous sampling site. Five different microbiota-samples were obtained from all stages; one oral swab, one skin swab (approximately 10 cm cranial to the pouch on the ventral surface, and moistened with sterile saline), one pouch swab, one cloacal swab and a fresh faecal pellet sample (when possible). Only cloacal samples from lactating females were included in the final analysis. From lactating females, a minimum of three milk drops was obtained – by stripping fingers along the length of the teat, from base to tip. Milk droplets were collected on a swab head. For joeys, depending on the size of the animal, three different samples were obtained: one oral swab, one skin swab (mid-abdomen) and one cloacal swab. Samples were collected by swabbing a given area with swabs moistened with a few drops of sterile saline for 5 s. Faecal pellets were collected straight from the cloaca when possible, or from the bottom of the hessian sack the animal had been kept in. The pellets were dissected by using a sterilized spatula to scoop out the centre of the pellet. The spatula was cleaned with 5% bleach and 80% ethanol between samples. Additionally, a ‘sample blank control’ (a swab held in the air for 30 s) was collected at the start of each day during sample collection. A new sterile saline tube was opened each day, and a saline sample blank (unused swab with saline) was collected. All samples (swabs and faecal pellets) were transferred to empty 2 mL LoBind Eppendorf tubes and immediately stored in a mobile freezer (− 20 °C) for the duration of the field trip. Samples were then transferred to a − 20 °C freezer at the University of Adelaide prior to laboratory work. After sample collection and once the animal had recovered from the anesthetic, wombats were returned to their site of capture and the same warren complex from where they had been found.

### DNA extraction

All DNA extractions were performed in freshly decontaminated Perspex hoods in a pre-PCR laboratory to prevent contamination with amplicons [16]. Both DNA from swabs and faecal samples were extracted using the ZymoBIOMICS DNA miniprep kit (ZR BashingBead™ Lysis Tubes) according to the manufacturer’s protocol with slight modifications: to reduce contamination, all buffers and tubes needed for the various steps were aliquoted prior to opening any sample tubes. For swab samples, step 12 (inhibitor removal) was skipped to minimise the loss of DNA and reduce exposure to potential contamination. To minimise batch effects samples were extracted in a random order, except for faecal samples, which were extracted in separate groups due to the potential for high-biomass cross-contamination [16, 27]. To account for laboratory related contamination, extraction blank controls from each extraction group was included and carried through to DNA sequencing. Beat beating was performed on a vortex using an adaptor for 10 min.

### Quantitative PCR

The absolute abundance of bacterial DNA in selected samples was quantified using qPCR targeting a universal region of the 16S rRNA gene. We used forward primer: F – 5′-ACTCCTACGGGAGGCAGCAGT-3′ and reverse primer: R – 5′-TATTACCGCGGCTGCTGGC-3′ [28]. The qPCR reaction consisted of 12.5 μL Brilliant II SYBR® Green QPCR MasterMix, 10.5 μL dH_2_O, 0.5 μL each of 10 μM forward and reverse primer and 1 μL of DNA. DNA was amplified using an initial denaturation at 95 °C for 10 min, followed by 40 cycles of denaturation at 95 °C for 30 s, annealing at 50 °C for 1 min and elongation at 72 °C for 45 s. Reactions were analysed on a QuantStudio™ 6 Flex Real-Time PCR System*.* A figure of the resulting ct values (measure of 16S rRNA gene copy number, low ct = higher biomass, vice versa) was made in R using ggplot2 [29].

### Amplicon library preparation and quantification

All samples were PCR-amplified and uniquely barcoded for High-Throughput Sequencing (HTS) using primers targeting the V4 region of the bacterial 16S rRNA gene [30]. We used forward primer: 515F (AATGATACGGCGACCACCGAGATCTACACTATGGTAATTGTG-TGCCAGCMGCCGCGGTAA) and barcoded reverse primer 806R (CAAGCAGAAGACGGCAT-ACGAGATnnnnnnnnnnnnAGTCAGTCAGCCGGACTACHVGGGTW TCTAAT) – the 12 n’s represent unique barcode sequences. The PCR reactions were prepared in a pre-PCR laboratory in a 5% bleached-cleaned and UV irradiated hood. Single reactions [31] of 2.5 μL X10 HiFi buffer, 0.1 μL Platinum™ Taq DNA Polymerase (ThermoFisher), 19.2 μL dH_2_O, 0.2 μL 100 mM dNTP mix, 0.5 μL each of 10 μM forward and reverse primer and 1 μL DNA. DNA was amplified using an initial denaturation at 94 °C for 3 min, followed by 35 cycles of denaturation at 94 °C for 45 s, annealing at 50 °C for 1 min, elongation at 68 °C for 90 s, with final adenylation for 10 min at 68 °C, in line with the Earth Microbiome Protocol [32]. Gel electrophoresis was carried out for each PCR reaction on a 3.5% agarose gel to ensure the samples contained library constructs of the desired length (~ 390 bp). For each sample, 1 μL amplified DNA was mixed into 199 μL Qubit® working solution (diluted Qubit® dsDNA HS Reagent 1:200 in Qubit® dsDNA HS Buffer) and quantified using a Qubit® 2.0 Fluorometer. Samples were pooled equimolar and cleaned using AxyPrep™ (Axygen) following the manufacturer’s instructions. Because negative controls contained little DNA, they were pooled separately and spiked into the final pool at a flat volume [16]. The final pool was quantified, and quality checked using an Agilent TapeStation. DNA sequencing was performed on an Illumina MiSeq (v2, 2 × 150 bp) at SAHMRI (South Australian Health and Medical Research Institute).

### Data analysis

The resulting DNA sequencing data were processed and analysed using the QIIME2 (v2019.10) bioinformatic pipeline [33]. Demultiplexed paired-end sequence reads were merged using vsearch [34] (the following parameters were used for merging; maximum number of mismatches in overlap: 1, minimum overlap length: 40), quality filtered, and denoised into amplicon sequence variants (ASVs) using the deblur [35] plugin (merged sequences were trimmed to 250 bp). Putative contaminants were identified using decontam’s prevalence-based method [36], and removed from the feature table. The feature tables were rarefied using the minimum number of sequences per sample for diversity analysis, i.e. 4512 sequences for the overview of all sample types, 5885 sequences for the pouch-focused analysis, and 5976 sequences for the joey, milk and maternal-pouch focused analysis. The ASVs were classified taxonomically using the latest SILVA [37] 16S rRNA gene V4 region classifier (version: 138–99–515-806). A denovo phylogenetic tree was constructed using the ‘qiime phylogeny align-to-tree-mafft-fasttree’ command. Alpha diversity was estimated using the richness diversity index. Beta diversity was estimated using the unweighted UniFrac [38] metrics and visualized with principal coordinate analysis (PCoA) plots. The hypothesis that samples within one group - i.e. lactating pouches vs pouch samples from other reproductive stages - were more similar to each other than to samples in other groups was tested with ANOSIM (based on 999 permutations). A heatmap was generated to classify the most abundant ASVs in the pouch, and a BLAST (Basic Local Alignment Search Tool) search against the NCBI nt database was carried out on the resulting sequences [39]. Figures were created using ggplot2 [29] in R studio [40].

## Results

### Dataset description

Twenty six female southern hairy-nosed wombats were captured, including 14 adult and 12 subadult animals, ranging in weight from 9.1 kg - 26.8 kg (Fig. 1; SI Table 1). To examine females and pouch young at different developmental stages, samples from 8 individuals and 1 joey were collected on the first trip in April 2019, 9 individuals and 1 joey were collected in August, and 9 individuals and 3 joeys were collected in October (Fig. 1). According to head length measurements (SI Table 1) and a growth rate equation estimated by [23]; Age = (Head length - 5.2) / (0.393 ± 0.26), the age of the joeys sampled were estimated to be ~ 168 days for the joey caught in April, ~ 28 days for the joey caught in August, and ~ 37, ~ 73 and ~ 78 days for the joeys caught in October.

A total of 104 microbial samples were collected from the 26 female SHNWs captured, including 26 pouch samples, 26 oral samples, 17 faecal samples, 17 skin samples, 9 joey samples (5 skin samples, 2 oral samples and 2 cloaca samples), 5 cloaca samples, and 4 milk samples.

A total of 130 samples were sequenced (104 biological, 26 negative control) on an Illumina MiSeq (2 × 150 bp), which yielded 12,313,465 paired end sequences, with an average of 94,718 reads per sample. After merging paired end reads and denoising with deblur (250 bp trim), 5265 ASVs were identified across the dataset.

### Determination of sample biomass and limit of detection using qPCR

**Fig. 3:**
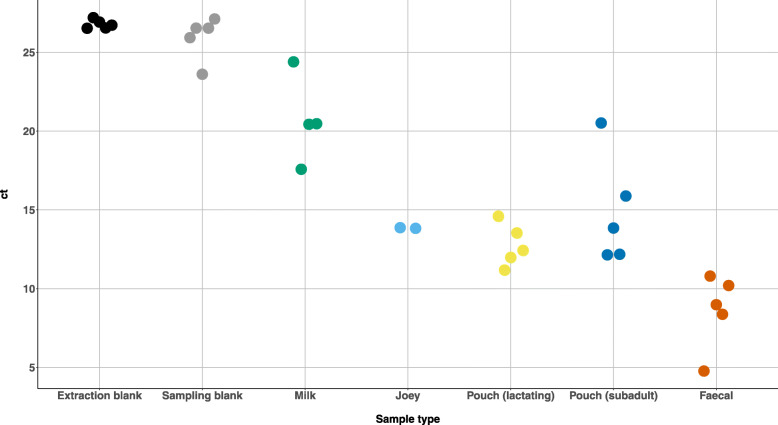
Determination of SHNW sample microbial biomass using quantitative PCR (qPCR) of the V4 region of the 16S rRNA gene. Y-axis values represent the cycle of threshold (ct) values for each sample, with lower ct values representing higher 16S rRNA gene copy number and higher microbial biomass, and vice versa. Joey = joey skin samples

Because 16S rRNA gene amplicon methods are susceptible to DNA contamination [15], it is important to determine the limit of detection by comparing biological samples to negative controls [16]. This is especially important when investigating new sample types such as the marsupial pouches in this study, where the microbial biomass has not been previously estimated. We tested whether the sample types collected in this study contained higher absolute amounts of microbial DNA compared to negative controls by using qPCR of the V4 region of the 16S rRNA gene. The faecal samples showed the lowest cycle threshold (ct) values (highest DNA concentration), with the extraction blank control and sampling blank controls having the highest ct values (Fig. 3). Milk, joey, and pouch samples had ct values lower than the negative controls, suggesting that biological samples contained microbial DNA at levels higher than the limit of detection of this study (ct of ~ 27). As expected, milk samples contained low quantities of DNA (ct values 17.6–24.4), as we were only able to collect ~ 3 drops per wombat. Overall, we conclude that SHNW pouch samples contain relatively high quantities of microbial DNA that is not a result of contamination from the sampling or laboratory environment.

### Decontamination of dataset using decontam

To explore and remove potential contaminant taxa from our dataset, we exported the feature (amplicon sequence variant) table from QIIME2 into the phyloseq R package [41] and identified putative contaminants using the prevalence-based method in decontam [36]. This approach exploits the widely observed signature that contaminant taxa are likely to have higher prevalence in negative control compared to biological samples. The score statistic ranges from 0 to 1 and based on a histogram of decontam scores we chose a decontam score threshold of 0.5 (SI figure 2), which resulted in 60 features being classified as contaminants (SI figure 3, SI Table 2). Using a threshold value of 0.5 will classify features as contaminants if they are present in a higher fraction of negative controls than biological samples. Taxa classified as putative contaminants include *Acinetobacter*, *Bradyrhizobium*, *Pseudomonas*, *Ralstonia*, and *Sphingomonas* (SI Table 2), taxa that have previously been reported in the negative controls of multiple studies [16]. We filtered these putatively contaminant features from our feature table prior to subsequent analyses.

### Microbial diversity and composition of southern hairy-nosed wombats

**Fig. 4:**
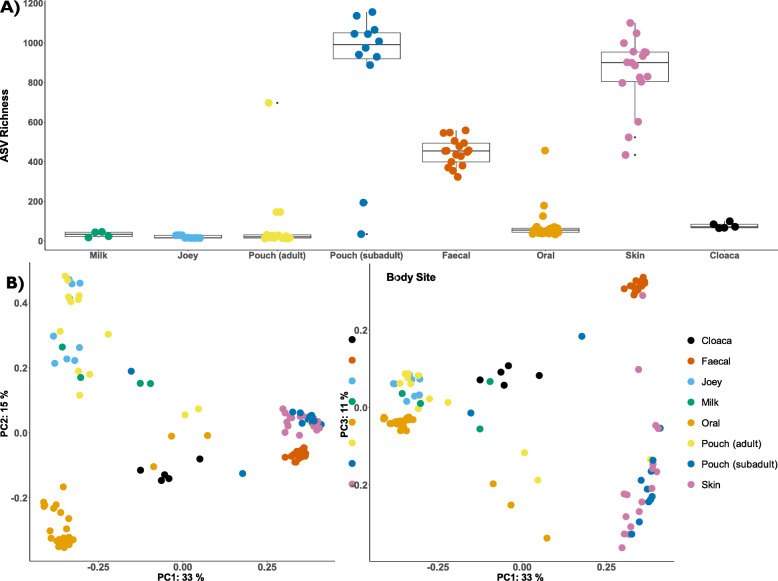
Overall microbial diversity comparisons of all remaining samples in the dataset after decontamination and filtering (*n =* 104), showing both oral (*n =* 26), skin (*n =* 17), cloacal (*n =* 5), faecal (*n =* 17), milk (*n =* 4), joey skin, oral, and cloaca (*n =* 9) and pouch samples divided into adult (*n =* 14) and subadult (*n =* 12) groupings. **a** Whisker-box plot showing the alpha diversity (richness) within each bodysite group. Horizontal lines in the boxes represent the median values; the lower and upper bound of boxes represent 25th and 75th percentiles, respectively. **b** Principal coordinates analysis (PCoA) plots based on the unweighted UniFrac distance matrix, portraying beta diversity between the different body site samples. The two plots display coordinates 1 versus 2 (left pane) and coordinates 1 versus 3 (right pane)

To provide context for the pouch samples, we sought to characterize the microbial diversity and composition of microbes at the different body sites collected. The skin and subadult pouch samples contained the highest microbial diversity (~ 900 features) when compared to other sample types (Fig. 4a, richness pairwise Kruskal-Wallis *p*-values < 0.05; SI Table 3). As expected, skin and subadult pouch samples contained similar levels of microbial diversity (Pairwise Kruskal-Wallis *p*-value > 0.05; SI Table 3) as subadult pouches are underdeveloped, open to the external environment, and generally resemble skin in appearance (Fig. 2a). Faecal samples contained the next highest level of microbial diversity (~ 450 features), followed by cloacal (~ 100 features) oral samples (~ 50 features). The reproductive samples (adult pouch, milk, and joey) contained the lowest microbial diversity (< 50 features). We found that subadult pouches contain significantly higher microbial diversity than adult pouches (Fig. 4a; pairwise Kruskal-Wallis richness *p*-values < 0.001; SI Table 3).

Analysis of microbial composition revealed that subadult pouch and skin samples clustered together (ANOSIM R = 0.003592, *p*-value = 0.425; Fig. 4b). Faecal samples formed a tight cluster of their own, separated from the subadult pouch and skin/subadult pouch samples across PC3 (ANOSIM R = 0.818670, *p*-value = 0.001; Fig. 4b). Faecal and skin/subadult pouch samples were separated from the oral and reproductive samples across PC1, which explained 33% of the variation (Fig. 4b; see SI Tables 4 & 5 for pairwise ANOSIM and permdisp tests). The oral and reproductive samples were separated along PC2 (ANOSIM R= > 0.75, *p*-values < 0.002; SI Table 4; Fig. 4b). Overall, we found statistically significant differences in microbial composition between body sites, with reproductive samples (adult pouch, milk, joey) clustering separate to other body sites sampled.

**Fig. 5:**
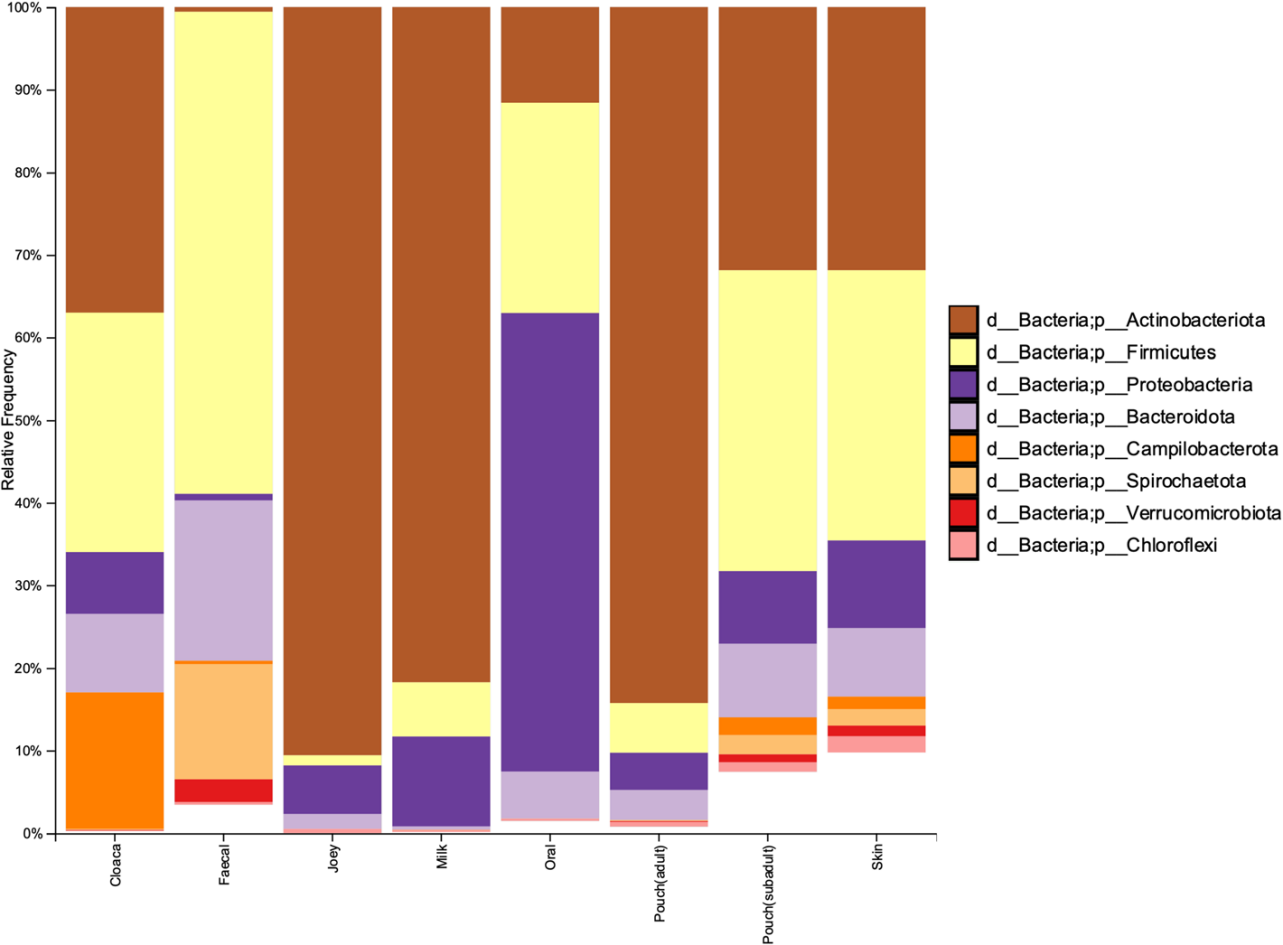
Bar chart showing the phylum-level grouped (mean) relative abundance of microbial communities found at the different SHNW body sites. Only the 8 most abundant phyla are displayed. See SI file 1 for the .qzv file containing taxonomy at different levels. Joey = joey skin, oral, and cloaca samples

Taxonomically, we found differences in the microbial communities between the body sites sampled. At the phylum level, faecal samples were dominated by Firmicutes (58.4%), Bacteroidota (19.4%), and Spirochaetota (14%) (Fig. 5). Within faecal samples, the most dominant families were *Christensenellaceae* (17.6%), *Spirochaetaceae* (13.9%), *Oscillospiraceae* (10%), *Rikenellaceae* (7.4%), and *Lachnospiraceae* (6.1%) (SI file 1). The Firmicutes to Bacteroidetes ratio in faecal samples was calculated to be 3.1:1 (SD = 1.1). In oral samples, the most abundant phyla were Proteobacteria (55.5%), Firmicutes (25.5%), and Actinobacteria (11.6%), with the most dominant families being *Pasteurellaceae* (26.5%), *Streptococcaceae* (16.3%), *Moraxellaceae* (14%), *Neisseriaceae* (9.4%), and *Micrococcaceae* (6.6%) (SI file 1). The most abundant phylum for reproductive samples (milk, adult pouch, joey) was Actinobacteriota (81.7–90.6%; Fig. 5), with the most abundant families being *Brevibacteriaceae* (20.9–36.5%), *Corynebacteriaceae* (12.4–22.2%), *Microbacteriaceae* (15–21.1%), and *Dietziaceae* (8.6–19%) (SI file 1). For the full taxonomy, both collapsed by sample type and at the individual sample level, see SI files 1 and 2.

### Effect of female reproductive state on pouch microbial communities

**Fig. 6:**
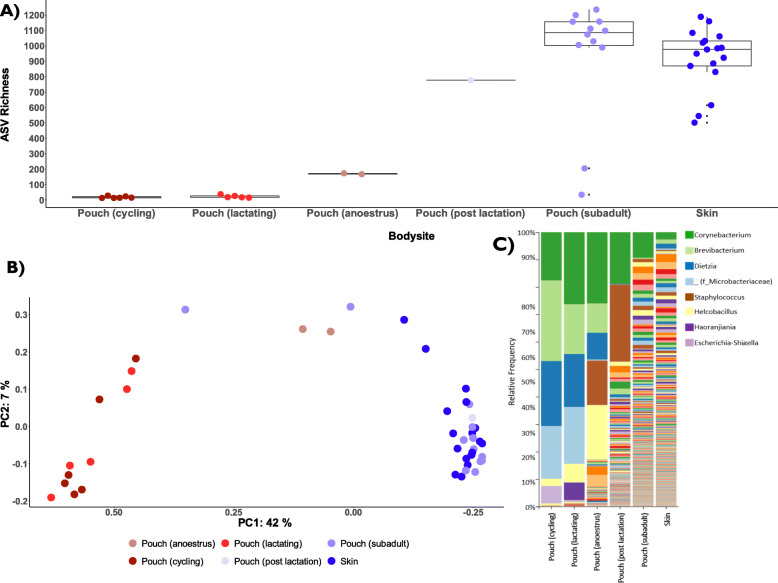
Diversity and composition of SHNW skin (*n =* 17) and pouch microbiome samples at different female reproductive stages: subadult (*n =* 12), cycling (*n =* 6), lactating (*n =* 5), post lactation (*n =* 1) and anoestrus (*n =* 2). **a** Whisker-box plot showing alpha diversity (richness) of the skin and the different pouch microbiomes. Horizontal lines in the boxes represent the median values; the lower and upper bound of boxes represents 25th and 75th percentiles, respectively. **b** Principal coordinates analysis (PCoA) plot based on the unweighted UniFrac distance matrix, portraying beta diversity between skin and the different pouch samples. **c** Bar chart showing the genus-level grouped (mean) relative abundance of microbial communities found at the skin and in the pouch during different reproductive stages. Only the nine most abundant genera are noted in the legend. One taxon could only be classified to family level (f_)

Given that the SHNW pouch undergoes both morphological and physiological changes throughout the reproductive cycle (Fig. 2), we next sought to test the hypothesis that changes in the reproductive cycle of female SHNWs influence the microbial diversity and composition of the pouch. To examine this, we filtered the feature table to only include pouch samples and their corresponding skin samples as controls. The data indicated that microbial diversity declined from reproductively inactive wombats (anoestrus, post-lactation, subadult) to reproductively active animals (cycling, lactating) (Fig. 6a). These differences were statistically significant for cycling and lactating vs. subadult wombats (richness pairwise Kruskal-Wallis *p*-values < 0.002 SI Table 6), although due to insufficient sample size we were unable to statistically test anoestrus (*n* = 2) and post-lactation females (*n* = 1). For microbial composition, we observed a similar trend across PC1 (42% of variation) that corresponded to female reproductive status (Fig. 6b), with cycling and lactating animals having statistically significant differences in microbial composition compared to subadult and skin samples (Unweighted UniFrac ANOSIM R values = > 0.91, *p*-values 0.001; see SI Tables 7 & 8 for all pairwise ANOSIM and perdisp comparisons). Taxonomically, the cycling and lactating pouch samples were dominated by the phylum Actinobacteriota, which contained five taxa that accounted for > 90% of the total relative abundance: *Corynebacterium*, *Brevibacterium*, *Dietzia*, *Microbacteriaceae,* and *Helcobacillus* (Fig. 6c).

### Characterisation of milk and joey samples

**Fig. 7:**
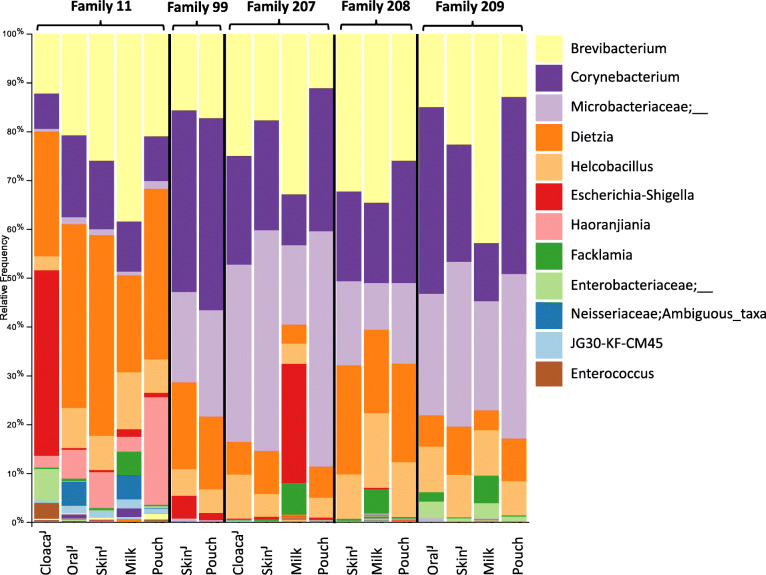
Bar chart showing the genus-level relative abundance of microbial communities found in the different types of SHNW joey samples (^J^), milk samples and the pouches of the mother. Each column represents a sample belonging to a specific individual. The family groupings include the samples from a mother and its joey. Estimated joey ages are ~ 168, ~ 28, ~ 37, ~ 73 and ~ 78 days, following the order of family groupings in the figure. The legend notes the twelve most abundant taxa, some of which could only be classified to family level

Next, we explored whether the age of pouch young influenced the microbial composition detected in the pouch or on the joey, and whether milk samples contained a distinct microbial signature. Samples were collected from five animals (trip 1: animal 11, trip 2: animal 99, trip 3: animals 207, 208, and 209) that had a pouch young with estimated ages of ~ 168, ~ 28, ~ 37, ~ 73 and ~ 78 days, respectively. The weights of the joeys ranged from ~ 16 to 500 g. Taxonomically, milk and joey samples resembled their corresponding pouches (Fig. 7). However, the taxonomic composition of microbes from family 11 (i.e. animal 11 and the corresponding joey samples) obtained from the most mature joey sampled, differed from the other, less mature joey families (Fig. 7). Samples from family 11 had a higher relative abundance of *Dietzia* and *Horanjiania*, and a lower relative abundance of *Microbacteriaceae*. The joey oral and milk samples from this animal both contained a *Neisseriaceae* feature that was also found in the oral sample taken from the mother (Fig. 7; SI figure 4). The joey cloaca sample also contained a higher abundance of *Escherichia-Shigella, Enterobacteriaceae, and Enterococcus*.

Finally, we sought to better classify the five most abundant microbes (accounting for > 90% of the relative abundance) in female wombats from cycling/lactating pouch, milk, and joey samples. We first created a heatmap of features from all cycling/lactating pouches, milk, and joey samples with minimum frequencies of 100 to determine the exact features that dominated these samples (SI figure 4). We then used BLAST (against the NCBI nt database) to find the closest reference alignment of these features (Table 1). Three of the five top features had best (or second-best for the g__*Helcobacillus*; s__uncultured_bacterium feature) hits to uncultured 16S rRNA gene clones isolated from the pouches of Tammar wallabies. One of the features, which was only classified to the family *Microbacteriaceae,* was highly divergent from any reference (239/250 nucleotide matches).

**Table 1:** | BLAST results from the top five most abundant features in the cycling/lactating pouch, joey, and milk samples. Two features have best hits, and one feature has multiple second-best hits to bacterial 16S clones isolated from Tammar wallaby pouches

FeatureID	SILVA_138 QIIME2 classified taxonomy	Sequence identity	Best hit
53fe3d733108e7d7b71644dd4f51b8b8	g__*Brevibacterium*; s__uncultured_bacterium	248/250	No best hit, multiple hits to *Brevibacterium* spp. and uncultured prokaryotes.
9532794e312626243216651ea0765320	g__*Dietzia*	247/250	Uncultured pouch clone 1530-P-3B from Chhour et al. 2010.
6fba0a71cfb9da18a3b61c9d3090de55	f__*Microbacteriaceae*	239/250	No best hit, multiple hits to *Gulosibacter* spp.
c0a3a4e78ac14be9f65454f6e10e163e	g__*Corynebacterium*	248/250	5 best hits to uncultured pouch clones from Chhour et al. 2010.
91548fb32f63077f9653391f4f1205a0	g__*Helcobacillus*; s__uncultured_bacterium	247/250, 246/250	Best hit (247/250) to a Hyena anal pouch clone, 3 s-best hits (246/250) to uncultured pouch clones from Chhour et al. 2010.

## Discussion

Because marsupials are born at a much earlier stage of development compared to eutherian mammals, the undeveloped immune system and developing microbiota of marsupial young are exposed to external environmental influences much earlier in their development [2, 42, 43, 11, 44]. This prompted us to investigate the microbial composition of both the pouch young and the pouch environment in the SHNW. However, DNA contamination of biological samples is increasingly being recognised as an important factor to control for in microbiome studies [15, 16]. This is particularly so for sample types that are novel or understudied, such as the pouch samples used in this study. Failure to control or account for DNA contamination can lead to erroneous biological interpretations [15, 16]. We thus sought to consider and control for DNA contamination by following guidelines proposed by Eisenhofer et al. [16]. We determined the limit of detection of our workflow by using qPCR, which enabled us to compare the amount of DNA in negative controls (sampling and extraction blanks) compared to biological samples. We found that the biological samples tested all had substantially higher concentrations of DNA than the negative controls, suggesting the presence of DNA in samples not derived from exogenous contamination. By including negative controls (sampling and extraction blanks), we were also able to explore the taxonomic profile of contaminants and distinguish them from those classified in biological samples. The taxonomic profile of negative controls was distinct from biological samples, containing taxa previously identified in the negative controls of other studies [15, 16]. We used decontam [36] to classify contaminant taxa using the prevalence method, which does so by identifying taxa that have higher prevalence in negative controls compared to the biological samples. We note that these precautions taken do not entirely eliminate the influence of DNA contamination, but nonetheless we are confident that the major biological signals observed in this study are not the result of DNA contamination. The workflow used also provides a solid framework for future investigations of the microbes associated with marsupial pouches, or other understudied body sites. Overall, this study represents the first contamination-controlled molecular investigation of microbes in the marsupial pouch.

As hypothesized, we found a significant overall decrease in microbial diversity in the pouches of reproductively active animals (gestating, near oestrus, or lactating). This finding is generally consistent with previous pre-NGS publications investigating the changing pouch microbiota of different marsupial species, such as the quokka [7, 8], tammar wallaby [10, 12], and the brushtail possum [11], but differs from a recent study in the Tasmanian devil [14]. Our findings regarding compositional changes between reproductive groups are also consistent with the aforementioned quokka and tammar wallaby studies [8, 12], where Actinobacteriota were found to be the most commonly identified phylum (> 80%) in lactating animals, with *Corynebacterium* as the dominant genus. In contrast, Peel et al .[14] identified *Pseudomonadaceae*, *Clostridiaceae*, and *Fusobacteriaceae* as the most abundant taxa in Tasmanian devil pouches. When comparing microbial patterns between different species, it is worthwhile noting differences in host species anatomy and behaviour. Marsupials from families such as Macropodidae and Dasyuridae are known to groom (by licking) the urogenital area and pouch prior to and after birth [18]. Conversely, the stocky anatomy of both the SHNW and koala, and their ventral/posterior-facing pouches would leave them physically incapable of grooming the pouch and joey [18]. This raises questions such as, are the oral microbes and/or saliva of pouch-grooming marsupials protective to the joey? Are the endogenous antimicrobial peptides and other defensive mechanisms stronger in marsupials that are unable to groom the pouch? One may expect to find increased potency of AMPs in marsupials that do not have anterior-facing pouches such as the burrowing bettong (*Bettongia lesueur*), koalas (*Phascolarctos cinereus* )[5], and bandicoots [18]. How grooming affects the microbiology of the marsupial pouch is worthy of more research.

In the pouches of reproductively active females, we found five taxa that accounted for > 90% of the microbial composition: *Corynebacterium*, *Brevibacterium*, *Dietzia*, *Microbacteriaceae*, and *Helcobacillus*. Interestingly, *Brevibacterium*, *Corynebacterium*, and *Micrococcus* have also been identified in the pouches of quokkas, tammar wallabies, and brushtail possums [8, 10, 11]*. Corynebacterium*, the most abundant taxon across all reproductive pouch stages, is a highly diverse genus, commonly isolated as a commensal colonizing the skin, mucus membranes, oral cavity, and digestive tract of eutherian mammals [45]. *Brevibacterium*, which we found to be the most abundant taxon in cycling wombat pouches and milk samples, is generally associated with milk products, but species of this genus are also known as commensals or opportunistic pathogens reported from other contexts, such a human skin [46, 47]. The genus *Dietzia* has been isolated from environmental, human, and animal samples [48], with few reports implicating it in human disease [49]. The family *Microbacteriaceae* comprises a large group of predominantly aerobic, Gram-positive bacteria that are found in various ecosystems associated with plants, fungi, animals, and clinical specimens [50, 51]. The genus *Helcobacillus* has only been described recently and consists of one named species which was isolated from a human skin infection [52]. BLAST searches of the most abundant features of these taxa revealed that three (*Corynebacterium*, *Dietzia* and *Helcobacillus*) of these five had closest hits to uncultured 16S rRNA gene clones isolated from the pouches of tammar wallabies [12]. This is interesting given that tammar wallabies and SHNW are allopatric and are thought to have shared a common ancestor ~ 50 million years ago [53]. One interpretation of this finding is that these taxa are host-associated commensals or symbionts that have diverged since the last common ancestor of tammar wallabies and SHNW. The amount of DNA sequence divergence between these three SHNW and tammar wallaby 16S rRNA gene V4 sequences is 2–4 nucleotides (0.8–1.6%), which is close to the 1–2% divergence per 50 million year estimate of 16S rRNA gene derived from insect endosymbionts [54]. Further research testing this idea should use full-length microbial 16S rRNA genes or whole genomes, and use samples from a wider range of marsupial species. Future identification of evolutionarily conserved, host-associated pouch microbes will help in determining whether such microbes provide benefit to marsupial reproduction.

We also attempted to see if we could study the development of the SHNW joey microbiota. The cloaca sample belonging to the most mature joey in the study (ID: 11 J) did show some potential gastrointestinal tract (GIT)-related microbiota differentiation (Fig. 7), such as the presence of the genus *Enterococcus*, which are common members of the GIT microbiota of other mammals [55]. We cannot rule out the possibility of it being a contaminant, but the finding is consistent with observations in a previous tammar wallaby study [12], where they sampled the entire GIT of the pouch young and detected two species of *Enterococcus* present throughout the GIT. We also detected *Enterobacteriaceae* in the two mothers accommodating the two most mature joeys in the study (family 11 and family 209) and in a faecal sample from a recently weaned juvenile (ID: 10). It was detected in highest abundance in the joey cloaca sample in family 11, but also in the oral and skin sample of the joey, and the pouch of the mother. In family 209, with a less mature pouch young, the highest abundance of *Enterobacteriaceae* was found in the milk sample and the oral sample of the joey, but was also detected in the pouch of the mother and skin of the joey (no cloaca sample had been taken from this joey). We also found a high relative abundance of *Escherichia-Shigella* in some of the reproductive samples (Fig. 7), especially in the cloaca sample taken from the most mature joey (ID: 11 J). However, we caution that this could be due to contamination, and suggest that further work on these samples using shotgun metagenomics could be used to test the authenticity of this assignment. Finally, we found some evidence for oral microbial development in the oldest joey (Fig. 7; ID: 11 J), with the joey oral sample containing the same feature (Neisseriaceae;Ambiguous_taxa) present in the mother’s oral sample. Overall, we found preliminary evidence for joey microbiota differentiation within the pouch. Future studies using time-series sampling of multiple individuals and shotgun metagenomics to track strains are required to better understand the development of marsupial microbiota within the pouch.

In recent years there have been calls for the conservation community to recognise the importance of host-associated microbiomes in animal health [56, 57]. While the gut is currently the most extensively researched microbiome in mammals, the microbial communities of the female reproductive system (milk, vagina, pouch, etc.) remain understudied. This is despite the fact that reproductive failure, including infertility and pregnancy loss, or poor health of young following birth, represent a substantial problem to many threatened species. Such research could be highly relevant for improving captive breeding programs and the successful reintroduction or maintenance of wild populations. None of the extant wombat species have been bred reliably in captivity and there is to date no successful breeding program for the critically endangered Northern Hairy-nosed Wombat [58]. There is currently very limited data available on the influence of captivity or domestication on the reproductive microbiome of any species, but disruptions in maternal microbiomes could be related to pregnancy complications and maternal, foetal, and neonatal health [59]. Although not a part of the reproductive tract, the marsupium (pouch) should be considered part of the marsupial reproductive system as most of the development of the marsupial neonate occurs at this site. It would therefore be worthwhile to compare the potential differences in pouch microbiomes between wild and captive held individuals as a way of investigating the correlation of reproductive success in the wild and failure in captivity. Additionally, using the SHNW as a research model for studies of this nature may provide valuable insights that could help with the conservation of the critically endangered Northern Hairy-nosed Wombat, which only has an estimated 100 mature individuals remaining [60].

## Conclusion

This study has generated the first baseline microbiota data for different body sites of wild Southern Hairy-nosed Wombats, including the first contamination-controlled investigation of the marsupial pouch microbiota. We presented a workflow that can control for DNA contamination and found that pouch microbial composition and diversity dramatically changes in relation to reproductive stage, suggesting a possible link to protection of pouch joeys during development. Further research is required to determine whether microbes present in the marsupial pouch are important to ensure normal development and survival of the joey and thus the reproductive success of marsupials.

## Supplementary Information

**Additional file 1 **SI figure 1: A Southern Hairy-nosed Wombat (*Lasiorhinus latifrons*) photographed at the study site. SI figure 2: Histogram of decontam scores. SI figure 3: Prevalence/prevalence plot of taxa identified in biological samples vs negative controls. Taxa identified as putative contaminants (> 0.5 decontam score) are coloured blue. SI figure 4: Heatmap of taxa found in pouch/joey samples.

**Additional file 2.**

## Data Availability

Demultiplexed DNA sequences are available at the NCBI’s SRA: PRJNA629644. SI file 1: 10.25909/5eb8b6001c15b SI file 2: 10.25909/5eb8b611d67eb
