# Supplementary figures and images for: Host reproductive cycle influences the pouch microbiota of wild southern hairy-nosed wombats (Lasiorhinus latifrons)
Source: Anim Microbiome. 2021 Jan 25;3:13. doi: 10.1186/s42523-021-00074-8 (PMC7836174; doi:10.1186/s42523-021-00074-8)

SI figure 1

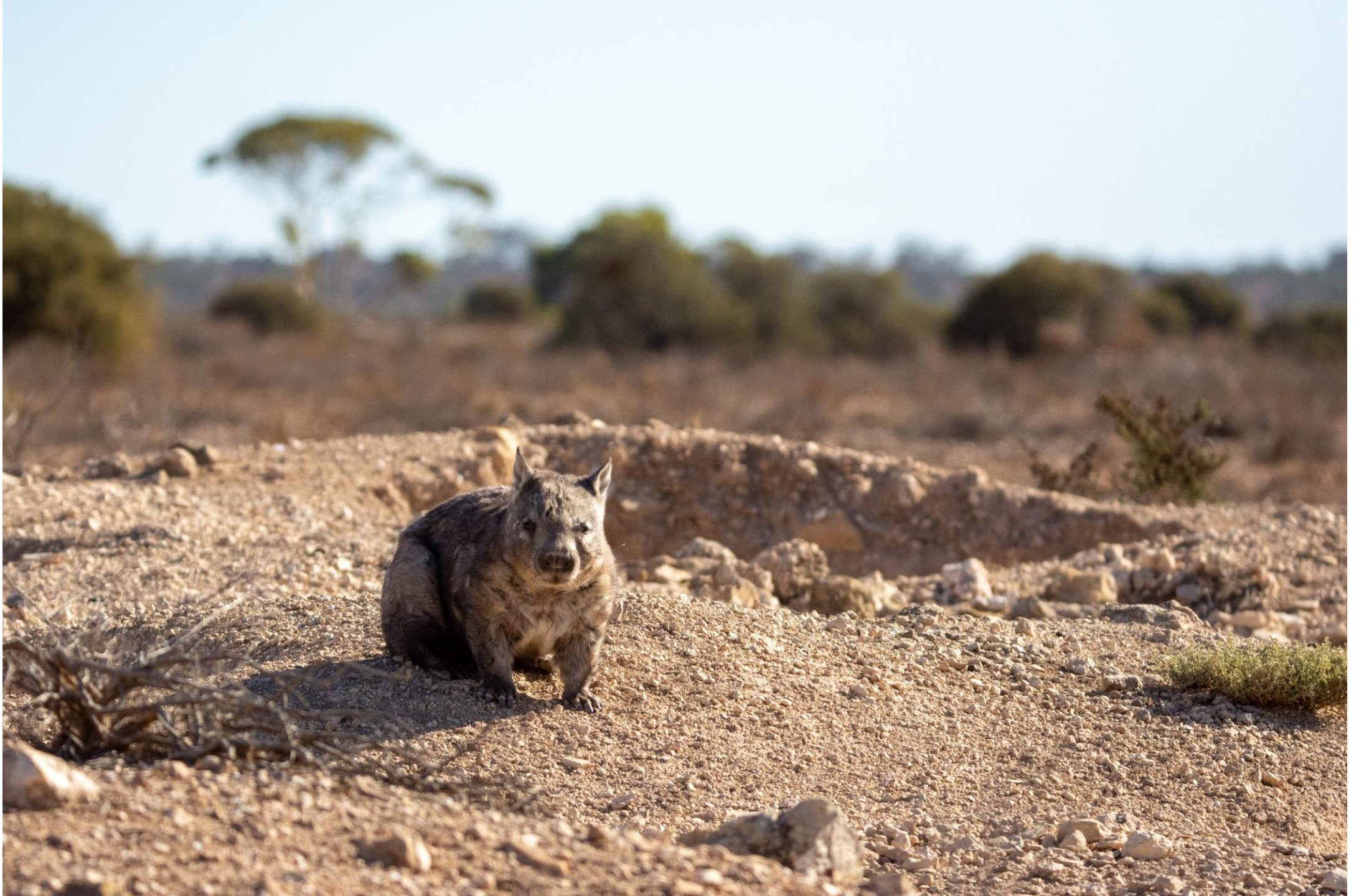

SI figure 2

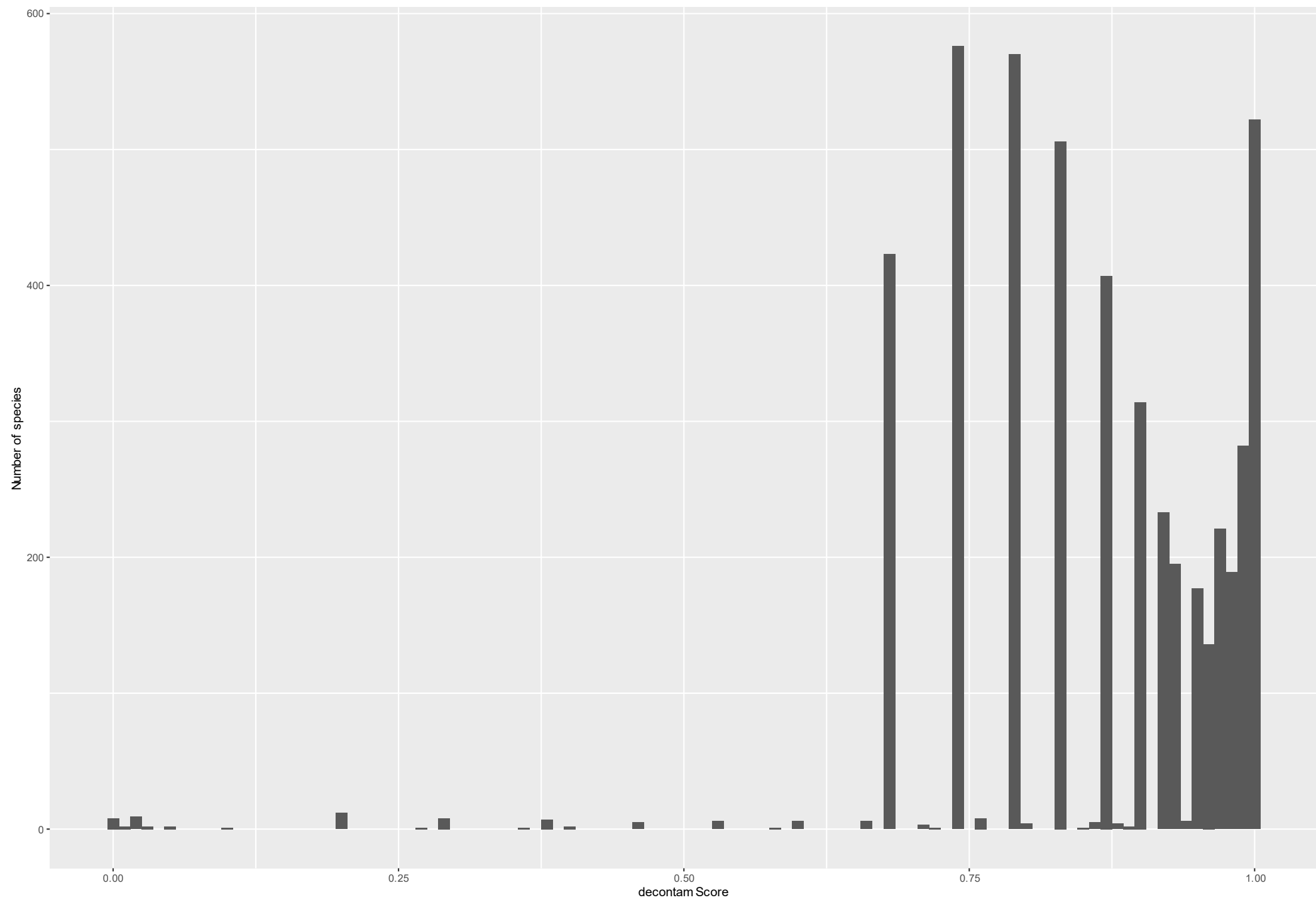

SI figure 3

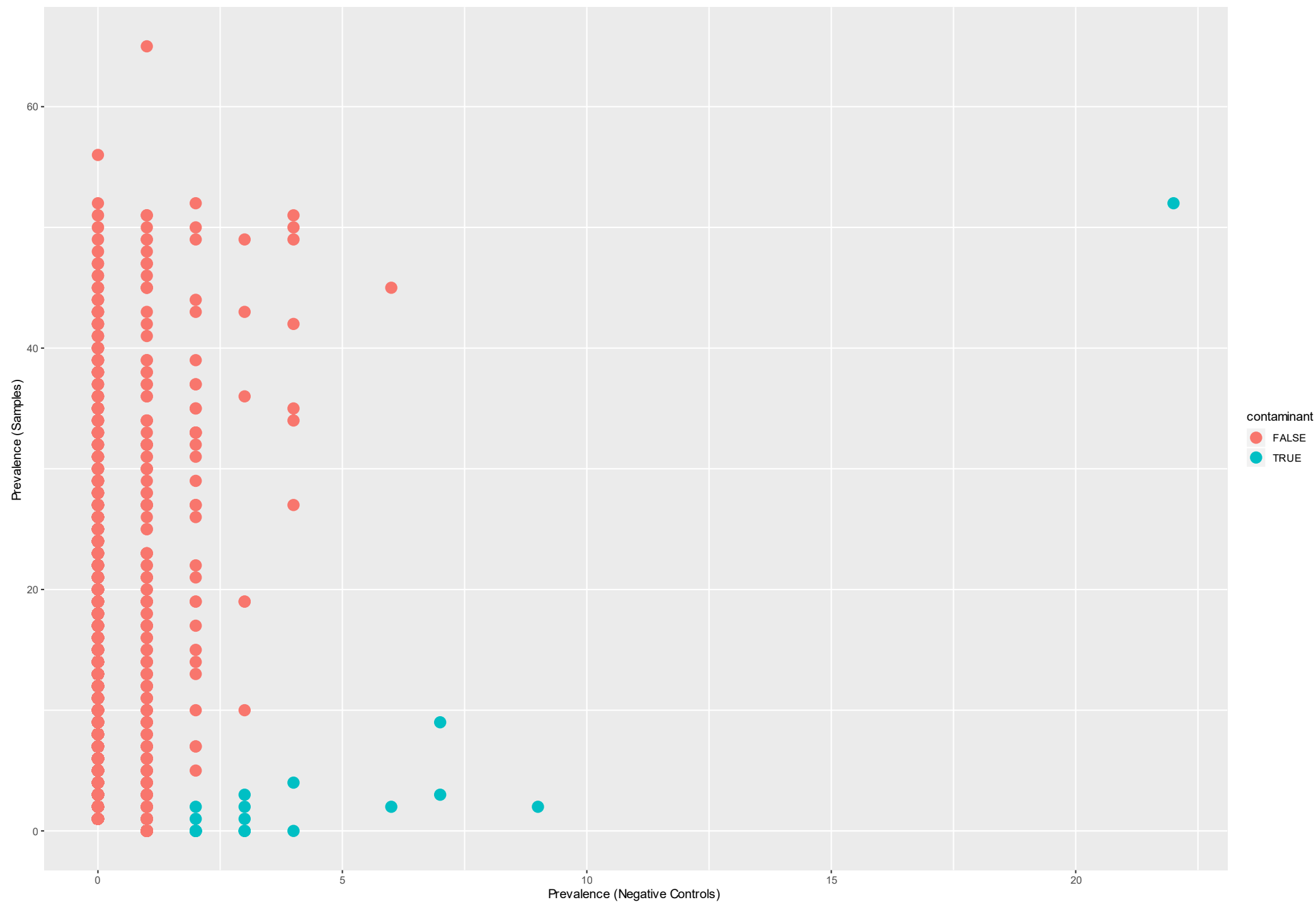

SI figure 4

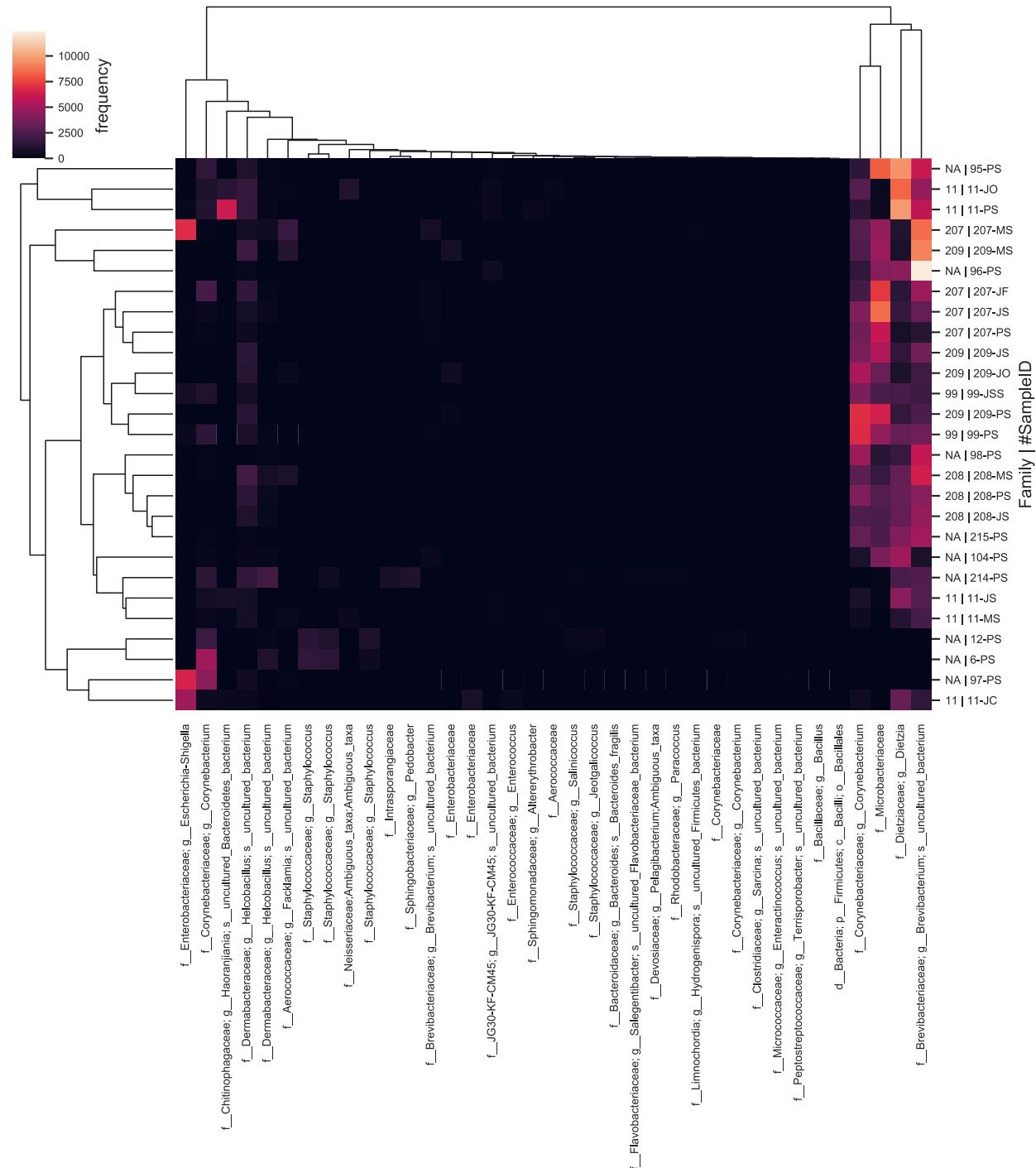

Supplement: Supplementary file 1 — Additional file 1 SI figure 1: A Southern Hairy-nosed Wombat (Lasiorhinus latifrons) photographed at the study site. SI figure 2: Histogram of decontam scores. SI figure 3: Prevalence/prevalence plot of taxa identified in biological samples vs negative controls. Taxa identified as putative contaminants (> 0.5 decontam score) are coloured blue. SI figure 4: Heatmap of taxa found in pouch/joey samples. [file 42523_2021_74_MOESM1_ESM.pdf]
